# The Conjusome—A Transient Organelle Linking Genome Rearrangements in the Parental and Developing Macronuclei

**DOI:** 10.3390/microorganisms11020418

**Published:** 2023-02-07

**Authors:** Christopher Janetopoulos, Karl J. Aufderheide

**Affiliations:** 1 Department of Cell Biology, School of Medicine, Johns Hopkins University, Baltimore, MD 21205, USA; 2 Total Experience Learning, Albright College, Reading, PA 19612, USA; 3 Department of Biology, Texas A&M University, College Station, TX 77843, USA

**Keywords:** IES, anlagen, p-granule, macronucleus, DNA elimination, epigenetics

## Abstract

The conjusome plays an important role in the conjugation events that occur in *Tetrahymena thermophila*. The conjusome appears in the anterior of conjugant pairs during the early stages of new macronuclei (anlagen) development. It lacks a membrane, and is composed of a network of fibrous, electron dense material, containing background cytoplasm and ribosomes. Several proteins localize to this organelle, including Pdd1p, a chromodomain protein that participates in the formation of chromatin-containing structures in developing macronuclear anlagen, and is associated with the elimination of specific germ-line sequences from developing macronuclei. Conjugants lacking the *PDD1* allele in the parental macronucleus do not show Pdd1p antibody staining in conjusomes. Investigations were performed using mutant cell lines, uniparental cytogamy and drug treatment, and show that the conjusome appears to be dependent on parental macronuclei condensation, and is a transitory organelle that traffics nuclear determinants from the parental macronucleus to the developing anlagen. These data, taken together with Pdd1p knockout experiments, suggest the conjusome is involved in the epigenetic phenomena that occur during conjugation and sexual reorganization. This is likely a conserved organelle. Conjusome-like structures were also observed in another Ciliate, *Stylonichia*. In general, conjusomes have features that resemble germ line P-granules.

## 1. Introduction

The dikaryotic nature of ciliophoran protozoa involves two distinct, differentiated nuclei, macronuclei (anlagen) and micronuclei, positioned side by side in a common cytoplasm. These two nuclei have very different structures, organizations and functions to the cell. The diploid micronucleus is mostly transcriptionally inactive, and during vegetative growth, its genome is in a transcriptionally incompetent state [1,2]. The micronucleus is analogous to the animal germ line, in that it is reserved to be a source of genomic materials during sexual events. The polyploid macronucleus is transcriptionally active, governing most of the phenotype of the cell, and is analogous to the animal soma line in that it is discarded during sexual events [3]. In *Tetrahymena,* sexual nuclear reorganization involves the condensation and eventual destruction of the existing parental macronucleus. The micronucleus undergoes a standard lower eukaryotic meiosis, followed by loss of all but one meiotic product, which then divides by mitosis. Reciprocal exchange of haploid gamete nuclei with the conjugation partner is followed by fertilization, producing a diploid zygote nucleus in each cell. Mitotic products of the zygote nucleus eventually differentiate into new macro- and micronuclei in each partner of the conjugation. The spatial signaling cues leading to this nuclear differentiation at the anterior and posterior of the cells have yet to be determined.

The micronuclear version of the genome consists of chromosomes in which the coding sequences of the genes are segmented by intervening non-coding sequences, the Internal Eliminated Sequences (IES) [4,5,6]. In the developing anlage, the chromosomes are fragmented and the IES are discarded. Selected remaining fragments are arranged into functional genes, capable of transcription, telomerized and amplified to produce a nucleus with a DNA content of approximately 45–60 C (with C being the amount of DNA in a haploid set of unreplicated chromosomes). These genomic rearrangments during macronuclear differentiation create expression-competent forms of the genes by deletion of the non-coding IES, followed by ligation of the coding sequences of DNA [7]. Approximately 15% of the unique micronuclear sequences are eliminated during this processing [8]. The pattern of programmed DNA elimination exhibits epigenetic inheritance, with the elimination of sequences dependent on the pre-existing rearrangements within the parental macronucleus [9,10,11]. *Tetrahymena* has been an important model system for investigating the mechanisms underlying this epigenetic transgenerational inheritance [12,13]. Since the parental macronuclear and micronuclear chromosomes never directly interact with one another, the molecular signals that mediate the processing in the developing macronuclei must be transmitted from the parental macronucleus to the new macronucleus through the cytoplasm.

Piwi-like small RNAs regulate the elimination of germline-limited sequences, including transposable elements, minisatellites and IESs during the development of the new macronucleus [14,15]. *Oxytricha* piRNAs act in the opposite way to the scnRNAs of *Paramecium* and *Tetrahymena*, retaining targeted sequences rather than eliminating them. The piRNAs in *Tetrahymena* resemble those of the germlines of animals, though in metazoans they often direct transcriptional silencing and heterochromatinisation of transposable elements [16]. In *C. elegans*, loss of the P-granules upregulates silencing by small RNAs against hundreds of endogenous germ line genes by pi-RNAs, suggesting that P granules protect germline transcripts from piRNA-initiated silencing [17].

We have previously reported the discovery of a unique organelle, the conjusome, which looks morphologically similar to P granules and appears during the time in conjugation when the developing macronuclei are differentiating [18]. Initially, the conjusome was found to contain the chromodomain protein, Pdd1p, which is also found in both parental macronuclei and developing anlagen. GFP-tagged versions also localize to the conjusome in a developmentally regulated manner [19]. The proteins encoded by the LIA (localized in macronuclear anlagen) genes, including Lia1, Lia3 and Lia5, also target the conjusome [20]. Of note, these proteins were expressed by targeting the constructs to the parental macronucleus, with the proteins initially expressed in the parental macronucleus and then in the conjusomes, followed by the developing anlagen. In other studies, *Tetrahymena* Chromodomain protein 1 (Tcd1) colocalized with histone 3 Lys-9 trimethylation (H3K9me3) and Pdd1p in the developing macronuclei and within the conjusomes [21]. These proteins are all strongly implicated in the regulation of the genome rearrangements, leading to the targeted elimination of germline-limited IESs, and are all apparently shuttling from the parental macronucleus to the conjusome and then the developing macronuclei, as initially proposed for Pdd1p [18]. The movement of molecules is not limited to proteins, as scan RNAs (scnRNAs) move both from the micronucleus to the parental macronuclei and from the parental macronuclei to the developing macronuclei [22,23,24]. Once in the new macronucleus, they guide heterochromatin formation and the methylation of histone H3 at lysines 9 and 27, and the accumulation of Heterochromatin Proteins (HP1) which contain chromodomains [25,26,27,28].

Here, we have analyzed the dynamics of conjusome activity by tracing the relative appearance and localization of Pdd1p in conjugating *Tetrahymena* using conjugation mutants and after different treatments that abort conjugation at various stages. The progress of the Pdd1p protein from one organelle to the next is remarkable in light of the newly emerging understanding of a molecular mechanism for the epigenetic process of macronuclear inheritance.

## 2. Materials and Methods

### 2.1. Cell Culture Conditions

Two genetically marked strains of *Tetrahymena thermophila* Nanny & McCoy, 1976, [CU 427 (Chx/Chx[cy-s]VI) and CU 438 (Pmr/Pmr[pm-s]IV)] were used for all experiments involving wild type cells. The genetically marked strains CU 427 (Mpr/Mpr[6-mp-s]VI) and CU 428 (Chx/Chx-[cy-s]VII) were used for all experiments involving the PDD1 gene knock out [29]. Defective “star” strains were of the A strain (III) [30]. Cells were grown to densities of 250,000–500,000 cells/mL in proteose peptone and yeast extract medium (PPY), and starved in Dryl’s solution [31] for 18–24 h prior to mixing. In order to induce cells to be sexually reactive, equal numbers of cells of complementary mating type were mixed following starvation. Mating efficiency (greater than 80–90% pairing) and kinetics similar to those previously described [32] were observed in all experiments. All time points refer to the time in hours from the mixing of complementary mating types.

### 2.2. Cytogamy and Environmental Shock

Uniparental cytogamy was induced by the procedure described by [30]. A hyperosmotic shock was delivered to mating cells by adding 150 μL of 20% glucose to 2.0 mL of mating cells (final concentration = 1.4%). After 45 min, the cytogamous conjugants were diluted first 1:10 in distilled water and subsequently resuspended into Dryl’s solution.

### 2.3. Drugs

Cycloheximide (Sigma) was administered at selected times during the conjugation at concentrations of 10 μg/mL, which completely blocks protein synthesis [33]. Aphidicholine (Sigma) was administered at selected times during conjugation at 10 μg/mL, inhibiting DNA replication as assayed by DAPI staining. More than 100 conjugants were examined for each condition.

### 2.4. Microcompression and Light Microscopy

Living, conjugating cells were picked individually at appropriate times with micropipette, and were placed into a microcompressor [34,35]. Immobilized, microcompressed conjugants were examined using high-resolution Nomarski D.I.C. or Zernike phase contrast optics on an Olympus BH2 microscope.

### 2.5. Immunofluorescence

All cells were prepared for immunostaining as previously described [36], with the following modifications: after fixation and resuspension in methanol, cells were washed twice in phosphate buffered saline (PBS) and then stored overnight in PBS with 2% bovine serum albumin (BSA). Cells were incubated for one hour the following day at room temperature with immune or preimmune rabbit serum (appropriately diluted in PBS with 2% BSA according to the titer of the immune serum). The cells were then washed twice in PBS followed by a one-hour incubation in Cy3-conjugated goat anti-rabbit serum (Sigma). For DNA staining, 0.1 mg/mL DAPI in distilled water was added to the cell suspension after immunolabeling, and the excess was then washed out with PBS before mounting. The specificity of the primary antibody to Pdd1p has been demonstrated in previous reports [37]. Labeled cells were resuspended in 150 μL of PBS, mounted on slides and examined using an Olympus BH2 microscope with an epifluorescence illumination system mounted with standard ultraviolet and green fluorescent protein filter sets.

## 3. Results

### 3.1. Analysis of Progeny Derived from Uniparental Cytogamy (UPC)

**Figure 1:**
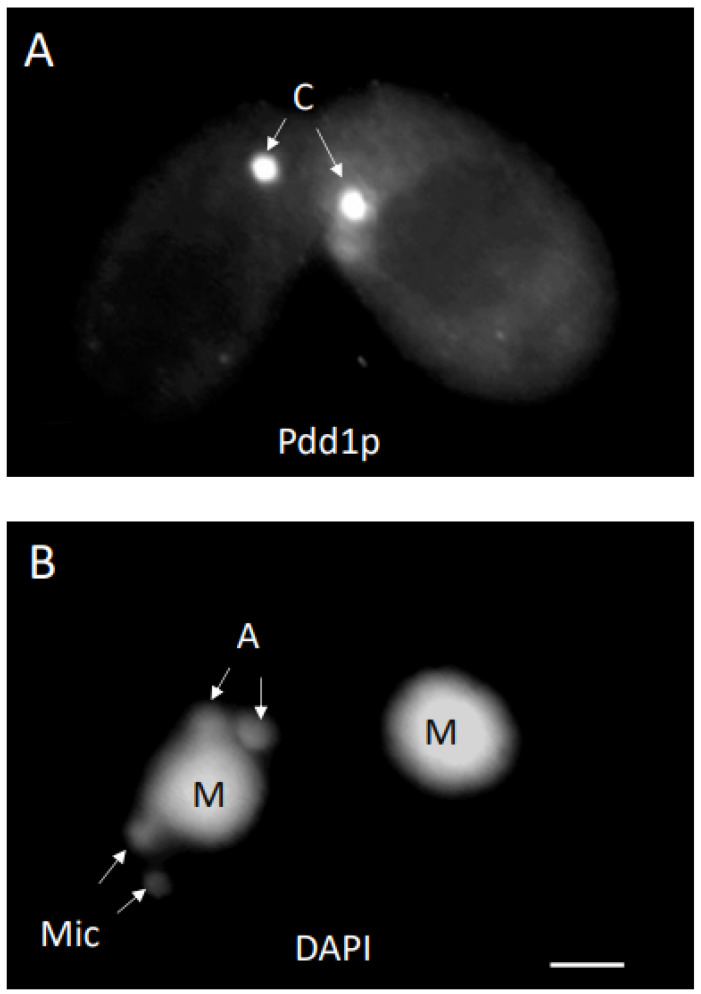
Conjugant pairs fixed at 7 h showed conjusome (C) staining to antibodies directed against Pdd1p in each cell (**A**). DAPI staining of the same pair revealed cytogamy had occurred in the left cell, while development was aborted in the right (**B**). The parental macronucleus (M) condensed in the star cell as well. Anlagen (A) are beginning to swell, while micronuclei (Mic) in the posterior are smaller. Bar is 5 micron.

We set out to examine the consequences of conjusome development under a variety of experimental conditions that altered conjugation events at various stages. In this UPC protocol, a wild-type strain was mated to a star strain partner. A star strain has a defective micronucleus, and is unable to contribute pronuclei for exchange and fertilization [30]. An osmotic shock was delivered at 5.75 h after mixing, which aborts the transfer of the pronucleus to the star strain, resulting in self-fertilization (cytogamy) in approximately 95% of the wild-type cells [30]. We specifically selected for pairs in which the wild type cell underwent cytogamy. Wild-type cytogamonts showed normal nuclear development; this included the appearance of conjusomes at the appropriate time (Figure 1A). In those pairs where the wild-type partner had undergone cytogamy, the star cell ceased nuclear development, as it had no pronucleus and did not receive one (Figure 1B). Two interesting features were apparent in the star cell partners: the existing parental macronuclei condensed even though no nuclear developmental program had been initiated; and conjusomes were present at the same time as in the wild type partner (Figure 1A,B).

### 3.2. Response of Cells to Cycloheximide or Aphidicholine Treatment

**Figure 2:**
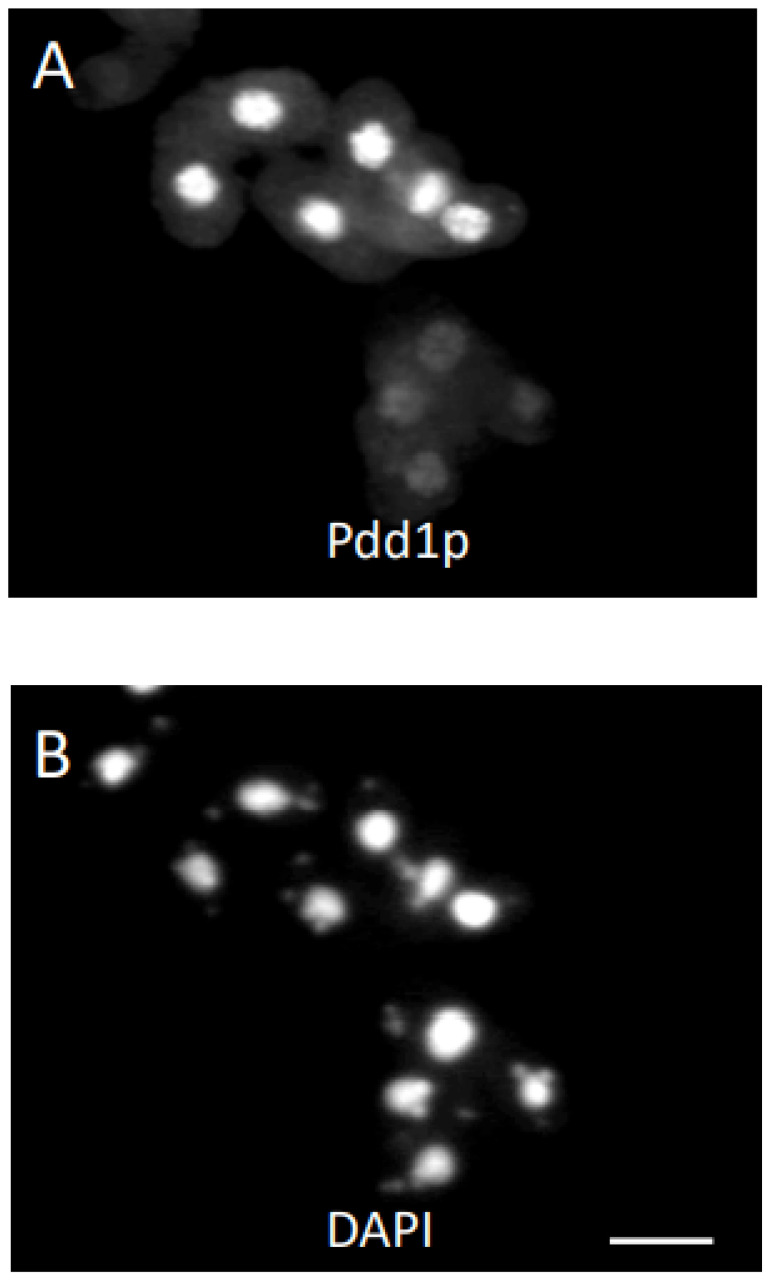
Multiple conjugant pairs treated at 6 h with cycloheximide and fixed at 8 h had the same Pdd1p staining pattern as 4.5 h treated cells that were fixed at 8 h (**A**). Only parental macronuclei stained positive for Pdd1p antibodies. DAPI staining revealed that cells completed meiosis (**B**). Bar is 20 microns.

In an effort to further elucidate the conjusome and the movement of Pdd1p between the organelles, various chemical treatments that affected nuclear divisions during conjugation were applied to cells. The protein synthesis inhibitor cycloheximide can block development in conjugation at various stages, presumably by inhibiting the synthesis of proteins necessary for various steps in development. Since Pdd1p is initially localized in the parental macronucleus, we blocked protein synthesis after Pdd1p should be present in the parental macronucleus, but before the appearance of the conjusome. DAPI staining of cells treated with cycloheximide at 4.5 h revealed that those cells rarely proceeded very far through meiosis, and often had only a single micronucleus (not shown). On the other hand, conjugants treated at 6 h often completed meiosis. Cells treated at 4.5 h and 6 h into conjugation produced similar results when analyzed by Pdd1p antisera. With more than 100 conjugants analyzed, greater than 75% showed heavy staining of the parental macronucleus with Pdd1p, no parental macronuclear condensation and no evidence of conjusomes (Figure 2A,B).

**Figure 3:**
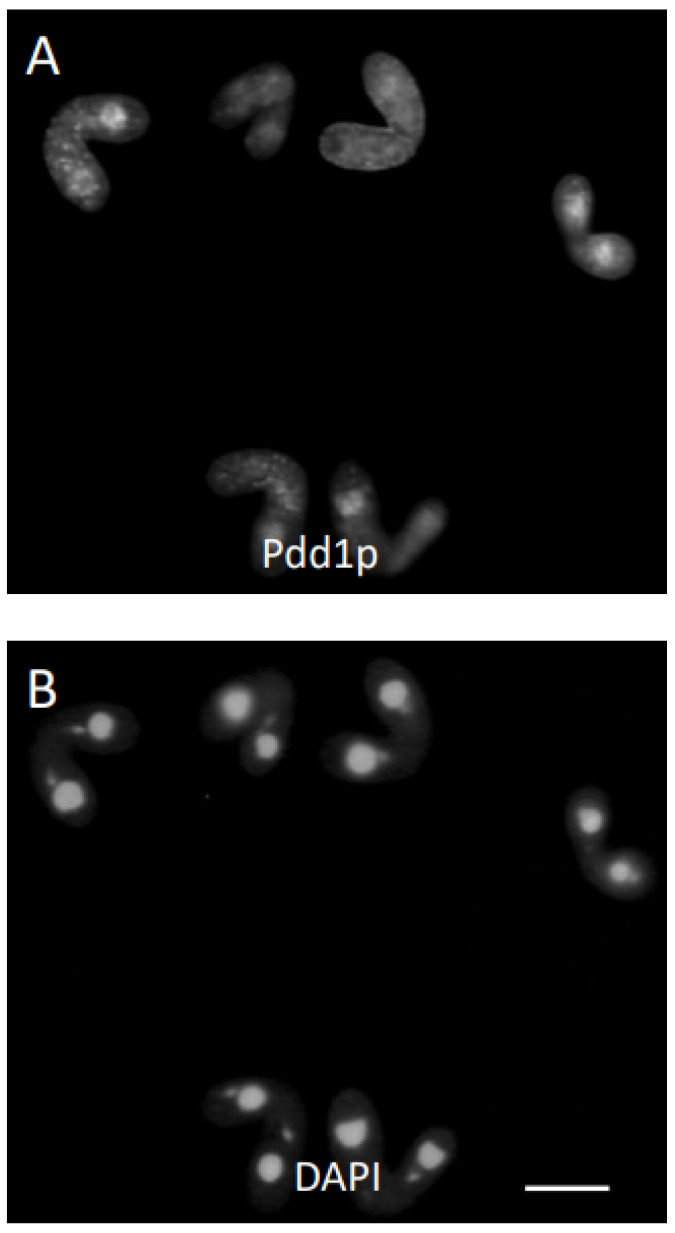

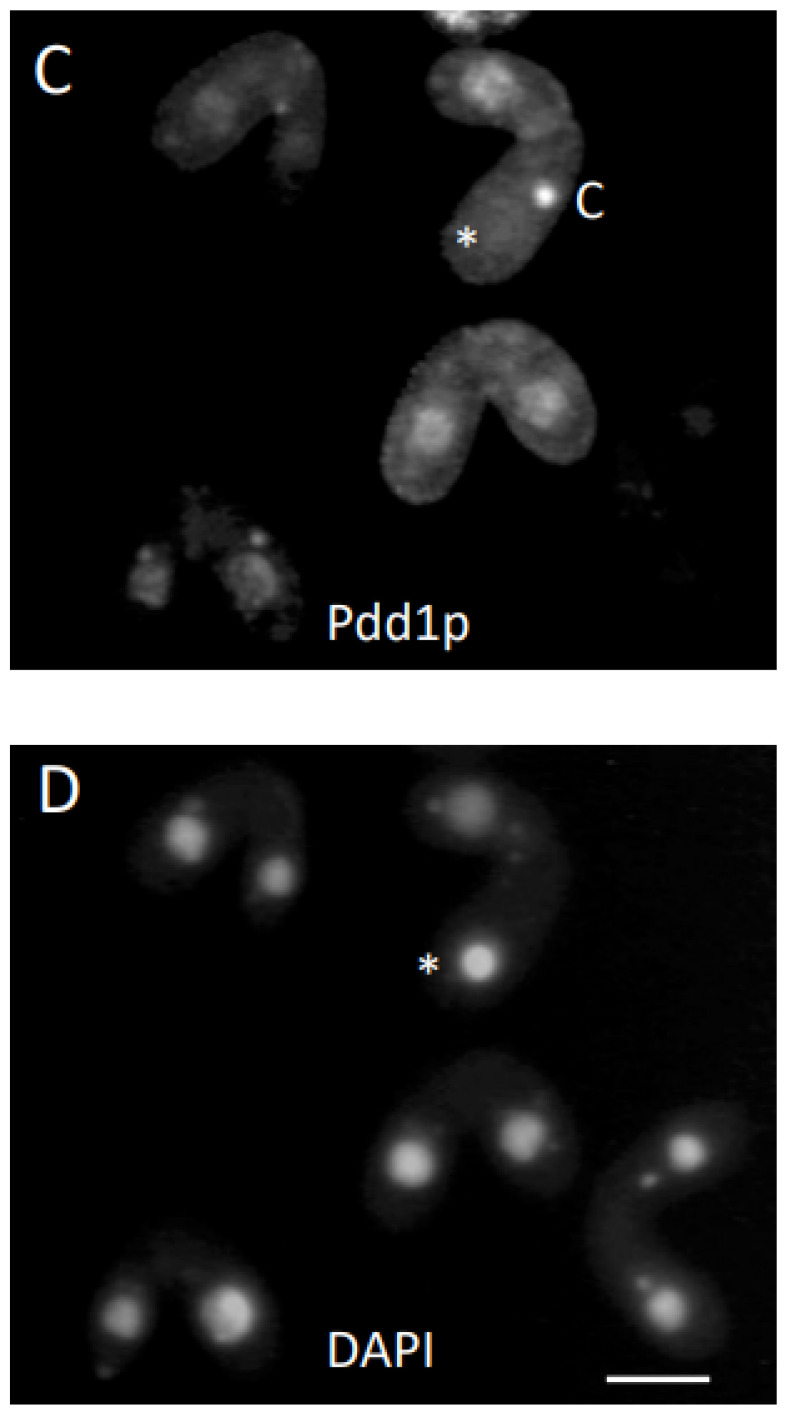
Conjugants treated with aphidicholine at 3.5 h looked remarkably similar to 4.5 h cycloheximide treated cells fixed at 8 h (**A**). DAPI staining revealed that the cells were blocked before meiosis (**B**). These cells were fixed at 6.5 h. Bar is 20 microns. Aphidicholine-treated conjugants fixed at 8 h often had localization of Pdd1p in both the parental macronuclei and conjusomes (C). Note the pair with the asterisk (**C**,**D**). The parental macronucleus has condensed and no longer stains for Pdd1p in the same cell which has a large conjusome (C). Bar is 20 microns.

**Figure 4:**
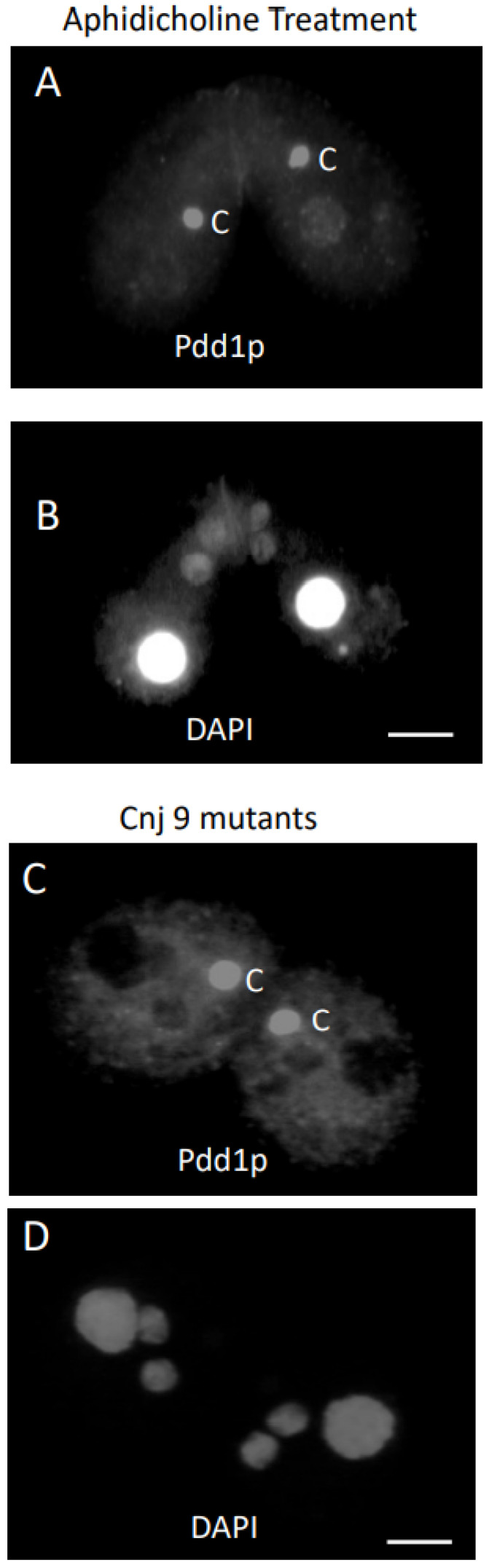
Cells blocked at the second postzygotic mitosis resemble the cnj9 strain when fixed at 9 h. Cells displayed a large conjusome (C) staining for antibodies directed against Pdd1p in each cell (**A**). The same pair stained for DAPI showed the two zygotic nuclei in each cell that failed to undergo the second postzygotic division (**B**). Bar is 5 microns. The cnj9 strain is blocked at the second postzygotic mitosis and showed a large conjusome (C) staining for antibodies directed against Pdd1p in each cell (**C**). The same pair stained for DAPI showed the two zygotic nuclei in each cell that failed to undergo the second postzygotic division (**D**). Bar is 5 microns.

Conjugant pairs were treated with aphidicholine at 3.5 h and 6.5 h after mixing. Pairs treated and stained at 3.5 h failed to complete meiosis, much like the cycloheximide pairs fixed at 4.5 h. The 3.5 h-treated cells, when fixed and examined at 6.5 h, were very similar to the 4.5 h cycloheximide-treated cells when examined at 6.5 h. Cells treated with Pdd1p antisera stained the parental macronuclei (Figure 3A,B). There were no conjusomes visible at this time. When 3.5 h treated cells were fixed and stained with Pdd1p antisera at 8 h, some cells (less than 10%) did have conjusomes. Interestingly, there was an inverse relationship between the intensity of Pdd1p staining in the parental macronucleus and the intensity in the conjusomes (Figure 3C,D). The parental macronucleus in this case had undergone condensation. Cells that completed the first postzygotic mitosis but were blocked at the second postzygotic division by aphidicholine had two diploid nuclei (Figure 4A,B). Interestingly, these conjugants looked very similar in nature to the *cnj9* mutant, which also fails to go through the second postzygotic mitosis (Figure 4C,D). We also examined the *cnj10* mutants [38] which showed a variety of nuclear defects. Conjusomes were not observed; these cells did not display staining for Pdd1p in any nuclei that appeared to be anlagen-like, and do not appear to undergo endoreplication as assayed by DAPI staining (not shown).

### 3.3. Knockouts Have Conjusomes

**Figure 5:**
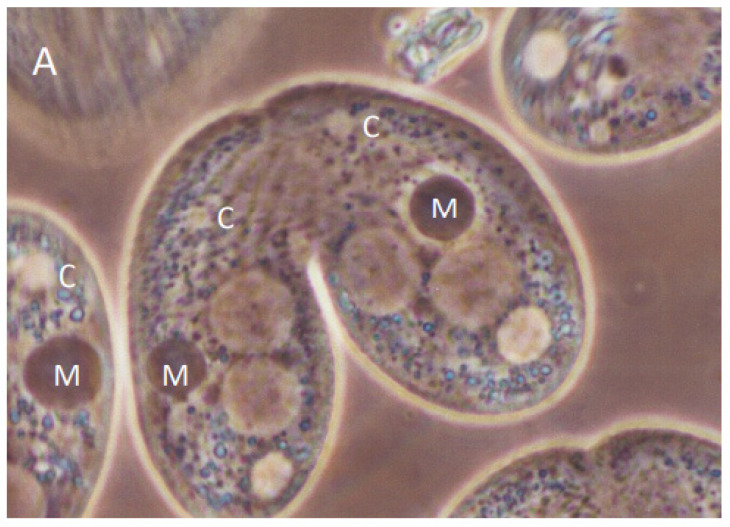

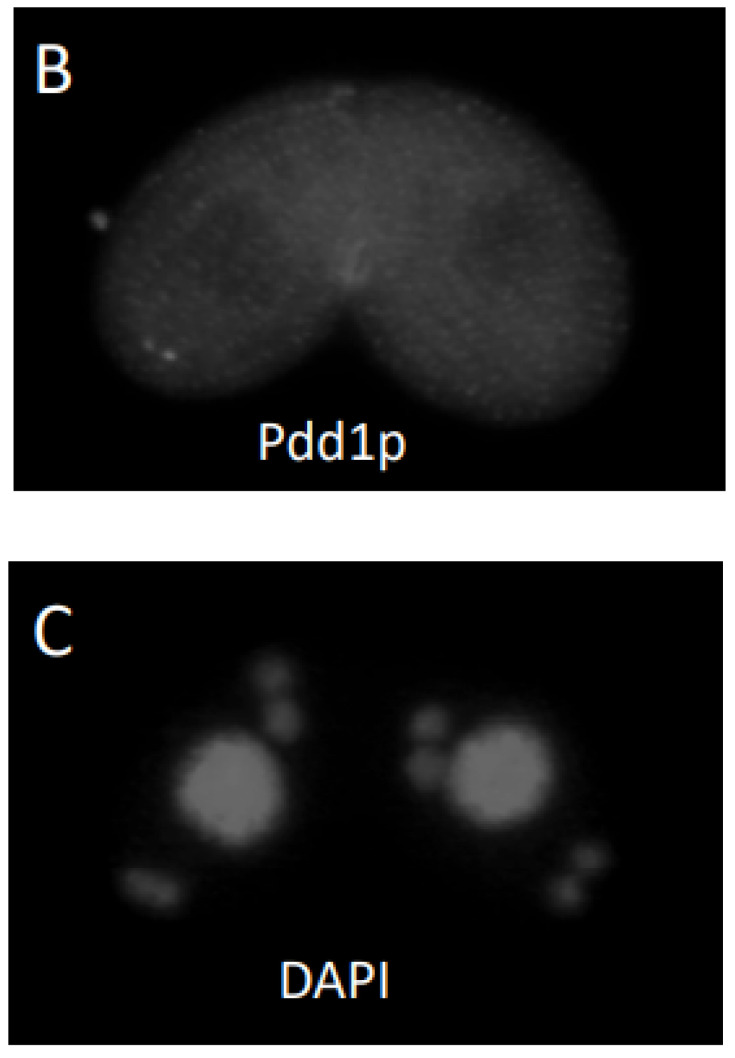

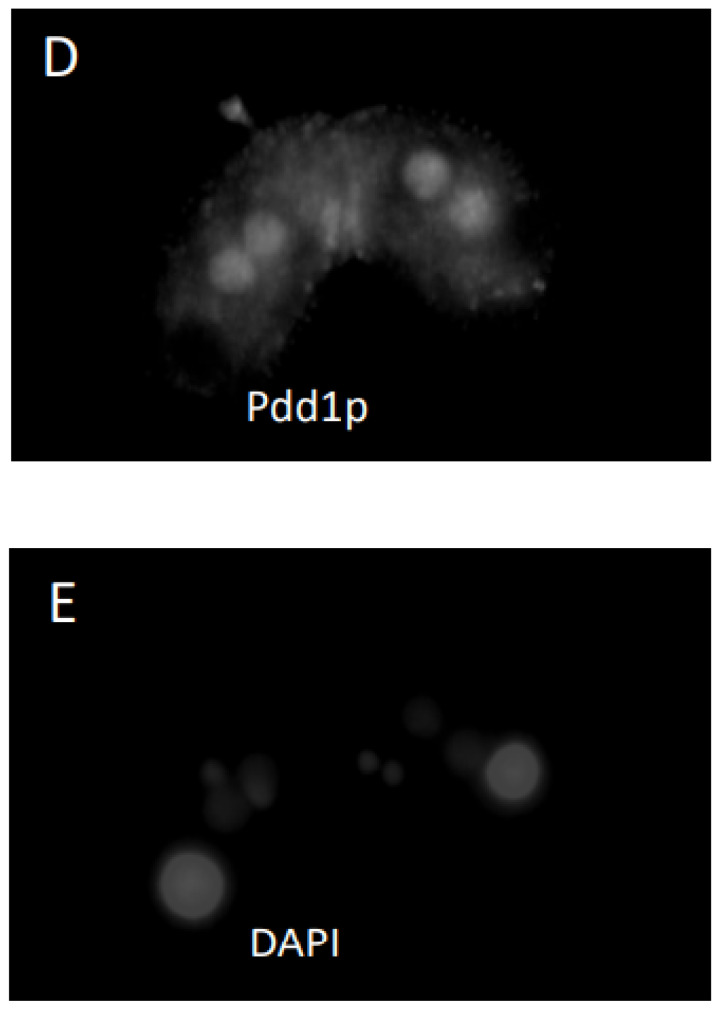
Phase contrast image of an 8 h conjugating pairs that had the PDD1 gene eliminated from the parental macrounucleus. The parental macronucleus (M) had condensed and conjusomes (C) were quite visible. Macronuclear anlagen were quite large at this time (**A**). PDD1 gene knockouts were examined using antibodies directed against Pdd1p at 7 h (**B**). Parental macronuclei and conjusomes did not show localization of Pdd1p. The same conjugant pair stained with DAPI showed that cells had just finished the last postzygotic division (**C**). PDD1 gene knockouts were examined using antibodies directed against Pdd1p at 9 h. Macronuclear anlagen showed localization to Pdd1p at the proper time (**D**). The PDD1 knockout is lethal if cells proceed through conjugation. The same pair is counterstained with DAPI (**E**).

The expression pattern of Pdd1p during conjugation has been documented in a number of previous reports [18,37,38,39,40,41]. Pdd1p appeared in the parental macronucleus early in conjugation, from 3 to 5 h. To investigate whether PDD1 transcription in the parental macronucleus was required for conjusome formation or for Pdd1p in the conjusome, we examined cells where the PDD1 gene was eliminated from the parental macronucleus, but not the micronucleus [29]. Living mutant conjugants were examined prior to examination of fixed specimens, and conjusomes were readily apparent by phase contrast optics while cells were under microcompression (Figure 5A). However, no Pdd1p staining was observed in any structures in the anterior of the cell lines containing the PDD1 gene disruption (Figure 5B,C) before 7.5 h. Conjusomes and parental macronuclei did not show Pdd1p staining. Pdd1p staining did appear at the proper time in the developing macronuclear anlagen as a consequence of expression of the *Pdd1* gene, derived from the germ line source (Figure 5D,E). This provided further evidence that the Pdd1p typically found in the conjusome is derived from the parental macronucleus.

### 3.4. Conjusomes Are found in Other Ciliates

We looked extensively for conjusomes in *Paramecium tetraurelia*, but they were not detected at the light level and Pdd1p antisera did not label nuclei or any other structures when examined by immunofluorescence. Interestingly, when we examined the hypotrich *Stylonychia lemnae*, these exconjugants showed staining of both the parental macronuclei, the developing macronuclei and conjusome-like structures (Supplementary Figure S1).

## 4. Discussion

### 4.1. Conjusomes Appear to Be Regulated by an Independent Pathway

In the UPC experiments reported here, the wild-type strain, containing a normal micronucleus, underwent cytogamy and development, while the star partner, containing a defective micronucleus, aborted sexual reorganization and further development. This experiment assessed whether conjusomes would be present if fertilization and development did not proceed normally in the star cell. The wild-type cell that underwent cytogamy showed what appeared to be a normal conjusome, as did the star partner. Interestingly, the parental macronucleus in the star partner condensed and became pycnotic, even though sexual reorganization did not occur. This data suggested that the signals required for both conjusome development and parental macronucleus condensation were independent of sexual reorganization and normal development. Furthermore, condensation of the parental macronucleus appears to be sufficient for development of the conjusome. It is not clear where the signal for parental macronuclear condensation originated, as cytoplasmic mixing between the wild type and star partner could have occurred.

To rule out cytoplasmic transfer of a signal from one cell to another, we attempted to block development in both conjugants. This was done by the addition of the DNA replication inhibitor aphidicholine to the conjugants at 3.5 h and 6 h. Conjusomes were sometimes present even though meiosis and fertilization did not take place. In some instances, the parental macronuclei condensed after aphidicholine treatment, as in the star strain. Like the two nuclei in the *cnj9* mutant that failed to develop and remained micronucleus-like, the conjugants treated with aphidicholine that failed to undergo the last postzygotic mitosis had two nuclei that were not positive for Pdd1p antibodies and had very large conjusomes, suggesting that those nuclei are either not competent to receive Pdd1p from the conjusome, or that there is a signal for molecules to move from the conjusome to the new macronuclear anlagen. The mutant *cnj10* contained no conjusomes at all. Remarkably, there did not appear to be any staining in the new macronuclear anlagen either. It is possible the *cnj10* mutation might be a defect in conjusome development, or there might be a defect that leads to the loss of Pdd1p after its localization in the parental macronucleus.

### 4.2. Pdd1p Is Trafficking between Nuclei

Aphidicholine-treated cells fixed between 6.0 and 10 h gave compelling data suggesting that Pdd1p is shuttling between old and new macronuclei, with the conjusome as the reservoir while the protein is in transit. In some pairs, there was a slight delay in the condensation of the parental macronucleus in one of the two cells of a conjugating pair. In such a case, the conjusome was larger in the cell that had a Pdd1p-negative, condensed macronucleus, suggesting shuttling of the Pdd1p from the parental macronucleus to the conjusome. Blocking protein synthesis during conjugation did not affect staining of Pdd1p in the parental macronucleus, though the parental macronucei did not condense, and there did not appear to be conjusomes. This suggests that continued protein synthesis is required for both condensation of the parental macronucleus and also the development of the conjusome.

Elimination of the *PDD1* gene from the parental macronucleus supported the hypothesis that Pdd1p is being transported from the parental macronucleus to the conjusome. The knockouts showed no staining of the parental macronuclei and no staining of the conjusomes, yet conjusomes were clearly still present. Staining of the developing macronuclei, which do contain the *PDD1* gene, did occur at the appropriate time.

### 4.3. Conjusomes Might Be the Vehicles of Epigenetic Information

Numerous labs have now reported seeing conjusomes by imaging fluorescently labeled proteins that are involved in the elimination of germiline limited sequences, including the Lia family of proteins and several chromodomain containing proteins critical for new macronuclear development. Using microcompression, we were able to clearly see these polarized organelles in living wild-type and *PDD1* null cell lines using phase contrast optics. This organelle has no membrane, and is likely similar in nature to other organelles like the P-granules, which are thought to be biomolecular condensates. There is some evidence mounting that the molecular glue holding these structures together could be RNA molecules [42]. The conjusome appears to be an important hub for moving proteins, but it is possible that non-coding RNAs containing information about which sequences to eliminate from the developing macronuclear genome also shuttle through the conjusome. Interestingly, the conjusome is found in at least one other Ciliate, *Stylonichia lemnae*, suggesting that this organelle has likely been overlooked in other protozoa.

How the conjusome assembles in the anterior of the conjugants and what role it plays in macronuclear determination have yet to be determined, but it is clear that many of the proteins involved in eliminating germ-line limited sequences pass through this structure. Work from many labs has shown the elegant molecular dance that the nuclei play during sexual reorganization, and the conjusome is yet one more partner demonstrating the remarkable ability of organelles to assemble in space and time within the cytoplasm and to signal to one another on cue. It is likely that the mechanisms regulating these processes will help in our understanding of other genome arrangements and in the regulation of similar organelles, like p-granules, which are critical for germ cell fate and occur in a wide range of species.

## Data Availability

For further supporting materials including microscopy data, contact the corresponding author or retrieve the publicly available dissertation of C.J. at https://oaktrust.library.tamu.edu/handle/1969.1/2 (accessed on 30 November 2022).

## Supplementary Materials

The following supporting information can be downloaded at: https://www.mdpi.com/article/10.3390/microorganisms11020418/s1, Figure S1: *Stylonichia lemnae* contain conjusome-like structures.
