# Supplementary material for: The Conjusome—A Transient Organelle Linking Genome Rearrangements in the Parental and Developing Macronuclei
Source: Microorganisms. 2023 Feb 7;11(2):418. doi: 10.3390/microorganisms11020418 (PMC9962563; doi:10.3390/microorganisms11020418)
Supplement: Supplementary file 1 [file microorganisms-11-00418-s001.zip › microorganisms-2102995-supplementary.pdf]

Conjusomes in Stylonychia

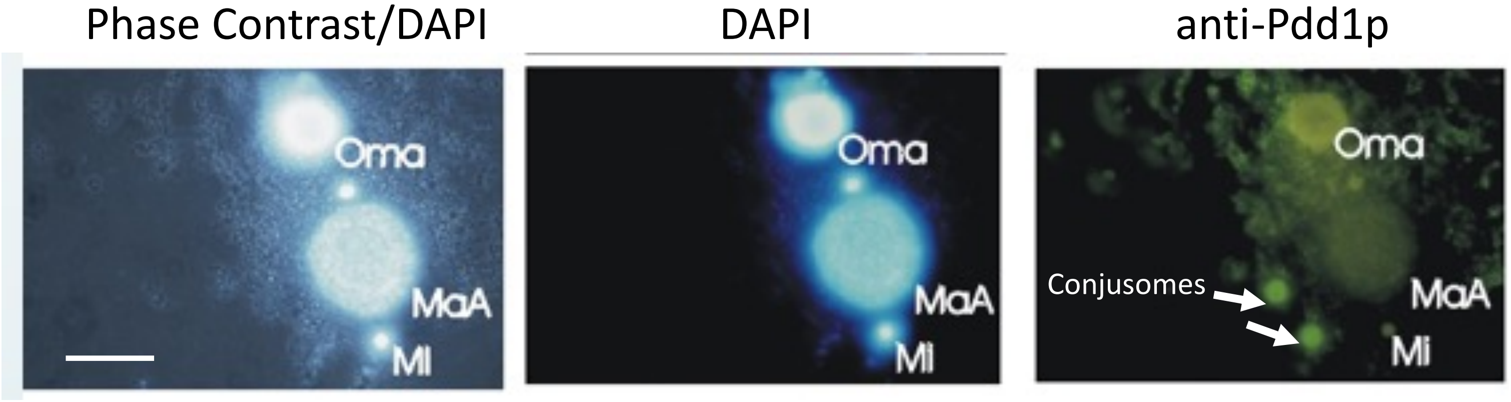

Supplementary Figure 1

Stylonichia lemnae contain conjusome-like structures. Overlay image of cells examined by phase contrast microscopy showing cell morphology and stained with DAPI to show the presence of DNA in the old macronucleus (Oma), the macronuclear analgen (MaA), and the micronucleus (Mi)(left panel); imaged with DAPI alone (middle panel); and imaged using antisera against Pdd1p (right panel). Conjusome-like structures are positive for Pdd1p, but contain no detectable DNA. Size bar equals 5 μm.
